# Functional Characterization of Waterlogging and Heat Stresses Tolerance Gene *Pyruvate* *decarboxylase* *2* from *Actinidia deliciosa*

**DOI:** 10.3390/ijms18112377

**Published:** 2017-11-09

**Authors:** Hui-Ting Luo, Ji-Yu Zhang, Gang Wang, Zhan-Hui Jia, Sheng-Nan Huang, Tao Wang, Zhong-Ren Guo

**Affiliations:** Institute of Botany, Jiangsu Province and Chinese Academy of Sciences, Nanjing 210014, China; m15105196421@163.com (H.-T.L.); wg20092011@163.com (G.W.); 13915954315@163.com (Z.-H.J.); qitianshengnan@163.com (S.-N.H.); wxtao@sina.cn (T.W.); zhongrenguo@cnbg.net (Z.-R.G.)

**Keywords:** kiwifruit, *Pyruvatedecarboxylase2* gene, waterlogging, heat stress, transgenic plants, environmental stresses

## Abstract

A previous report showed that both *Pyruvate* *decarboxylase* (*PDC*) genes were significantly upregulated in kiwifruit after waterlogging treatment using Illumina sequencing technology, and that the kiwifruit *AdPDC1* gene was required during waterlogging, but might not be required during other environmental stresses. Here, the function of another *PDC* gene, named *AdPDC2*, was analyzed. The expression of the *AdPDC2* gene was determined using qRT-PCR, and the results showed that the expression levels of *AdPDC2* in the reproductive organs were much higher than those in the nutritive organs. Waterlogging, NaCl, and heat could induce the expression of *AdPDC2*. Overexpression of kiwifruit *AdPDC2* in transgenic *Arabidopsis* enhanced resistance to waterlogging and heat stresses in five-week-old seedlings, but could not enhance resistance to NaCl and mannitol stresses at the seed germination stage and in early seedlings. These results suggested that the kiwifruit *AdPDC2* gene may play an important role in waterlogging resistance and heat stresses in kiwifruit.

## 1. Introduction

Plants have to cope with soil waterlogging or complete flooding during their lifetime, and flooding conditions impose a variety of challenges on the plant. Oxygen deprivation acts as the primary signal in response to flooding [1], and a lack of oxygen causes a reduction in respiratory efficiency. Therefore, adenosine triphosphate (ATP) synthesis is mostly provided by glycolysis coupled with nicotinamide adenine dinucleotide (NAD) regenerative pathways under anoxia including alanine production and ethanolic fermentation [2]. *Pyruvate decarboxylase* (PDC, EC 4.1.1.1) catalyzes the first step, which is responsible for the irreversible conversion of pyruvate to acetaldehyde. Alcohol dehydrogenase (ADH, EC 1.1.1.1) then converts acetaldehyde to ethanol, with the concomitant regeneration of NAD^+^ [2]. The overexpression of either *Arabidopsis PDC1* or *PDC2* results in improved plant survival in hypoxic conditions [2]. Promoter sequences of hypoxia-induced genes including *ADH, PDC*, and *Sucrose synthase* (*SUSY*), were analyzed in several plant species, and the results showed that the GT-box (contains a central motif GGTT) and GC-box (GGGCGG or its atypical hexanucleotide sequence) motifs were the DNA elements putatively responsible for hypoxia inducibility [3].

Previously, we reported that two out of three kiwifruit *ADH* genes and both *PDC* genes were significantly upregulated in roots following waterlogging treatment, suggesting that ethanolic fermentation was activated in kiwifruit roots following waterlogging treatment [4]. To understand the functions of the *AdADH1*, *AdADH2*, and *AdPDC1* genes, transgenic *Arabidopsis* overexpressing kiwifruit *AdADH1*, *AdADH2*, or *AdPDC1* were generated, respectively. The transgenic lines had higher waterlogging stress tolerance than the wild type (WT) after two weeks of waterlogging stress, suggesting that the overexpression of kiwifruit *AdADH1*, *AdADH2*, or *AdPDC1* in *Arabidopsis* enhanced waterlogging stress tolerance [5,6]. However, no other kiwifruit *PDC* genes have been characterized.

Extensive agricultural losses worldwide have been attributed to abiotic stresses such as waterlogging, drought, salinity, low temperature, and heat. Understanding the gene function of plant stress responses to extreme environmental factors is therefore of basic and practical importance [7,8]. In this paper, we present results demonstrating the function of another kiwifruit *PDC* gene named *AdPDC2* during waterlogging and other environmental stresses. The complete coding sequence (CDS) was isolated from *Actinidia deliciosa* (Jinkui) according to the sequence comp110797-co-seq1 obtained by Illumina sequencing [4]. Expression levels of *AdPDC2* were determined in kiwifruit after treatment with waterlogging, salinity, low temperature, heat, drought, and abscisic acid (ABA) using real-time quantitative reverse transcription polymerase chain reaction (qRT-PCR). Transgenic *Arabidopsis* lines overexpressing kiwifruit *AdPDC2* were generated to study the function of the *AdPDC2* gene in *Arabidopsis*.

## 2. Results

### 2.1. Isolation and Expression Analysis of AdPDC2

A previous report showed that both *PDC* genes were significantly upregulated in kiwifruit after waterlogging treatment using Illumina sequencing technology [4], and the kiwifruit *AdPDC1* gene function was analyzed [5]. In this study, another *PDC* gene, named *AdPDC2*, was cloned from *A. deliciosa* (Jinkui) according to the sequence (comp110797-co-seq1). The CDS contained a 1746 bp open reading frame (ORF), which encodes a protein of 581 amino acids with a predicted molecular weight of about 62.59 kDa and an isoelectric point (PI) of 5.86. Nucleotide sequence alignment showed that *AdPDC2* shared 67.4%, 67.3%, and 69.6% sequence identity with *AtPDC1* (GenBank accession No. NM_124878), *AtPDC2* (GenBank accession No. NM_119461), and *AdPDC1* (GenBank accession No. KU095879) (Figure S1), respectively. Amino acid sequence alignment showed that AdPDC2 shared 75.8%, 76.8%, and 77.7% sequence identity with AtPDC1 (GenBank accession No. NP 200307), AtPDC2 (GenBank accession No. NP 195033), and AdPDC1 (GenBank accession No. ALX37952) (Figure S2), respectively.

**Figure 1:**
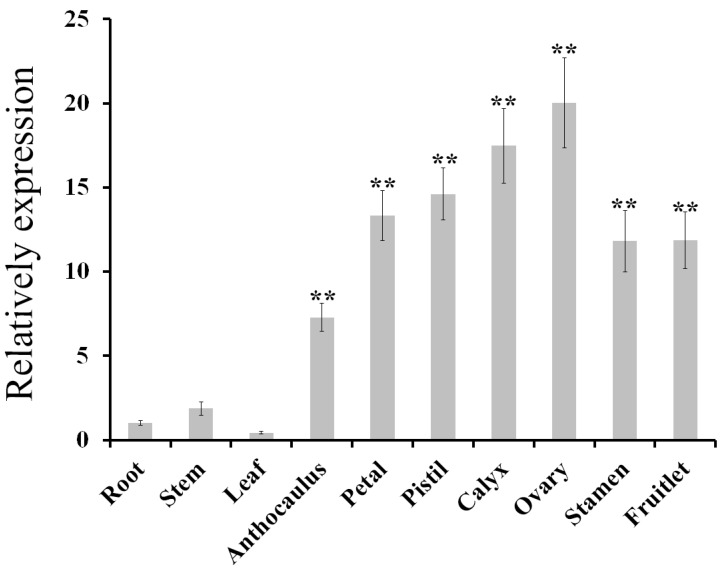
Expression pattern of *AdPDC2* in different kiwifruit (*A. deliciosa* ‘Jinkui’) plant tissues, including root, stem, leaf, anthocaulus, petal, pistil, calyx, ovary, stamen, and fruitlet (20 days after full blossom, DAFB) was assessed using qRT-PCR. *AdActin* was used as an internal control. Each column is the average of three replicates and each replicate has five plants. Bars indicate standard deviation (SD). Double asterisks indicate significant differences compared with root values (** *p* < 0.01).

**Figure 2:**
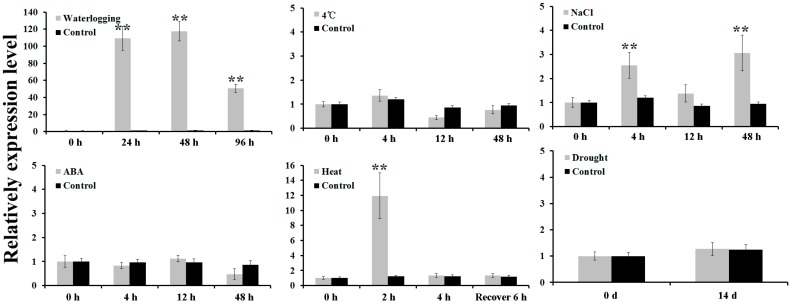
Expression patterns of *AdPDC2* in roots under waterlogging stress and in leaves under NaCl (200 mM), 4 °C, ABA (0.01 mM, Sigma, St. Louis, MO, USA), heat (incubated at 48 °C for 2 h and 4 h, then subsequently at 23 ± 1 °C for another 6 h), drought stresses (watering was withheld 14 d), and corresponding non-treatment plants (control) were assessed using qRT-PCR. *AdActin* was used as an internal control. Each column is the average of three replicates and each replicate has 15 plants. Bars indicate SD. Double asterisks indicate significant differences compared with non-treatment (0 h) values (** *p* < 0.01).

The expression patterns of *AdPDC2* in different organs of *A. deliciosa* and *A. deliciosa* subjected to a range of abiotic stresses were performed using qRT-PCR. The expression levels of *AdPDC2* in the reproductive organs (anthocaulus, petal, pistil, calyx, ovary, stamen, and fruitlet) were much higher than those in the nutritive organs (leaf, stem, and root) (Figure 1). *AdPDC2* expression was induced at 24 h, up to the highest point at 48 h, then decreased in *A. deliciosa* root after treatment with waterlogging (Figure 2). For NaCl stress, the expression of *AdPDC2* fluctuated during the first 48 h of treatment, and the *AdPDC2* transcript was significantly induced at 4 h and 48 h (Figure 2). The *AdPDC2* transcript was increased significantly at 2 h after treatment with heat, then decreased to a low level (Figure 2). ABA, low temperature, and drought stresses could not induce the expression of *AdPDC2* in *A. deliciosa* at the given time points (Figure 2).

### 2.2. Overexpression of AdPDC2 Enhances Tolerance to Waterlogging Stress in Arabidopsis

**Figure 3:**
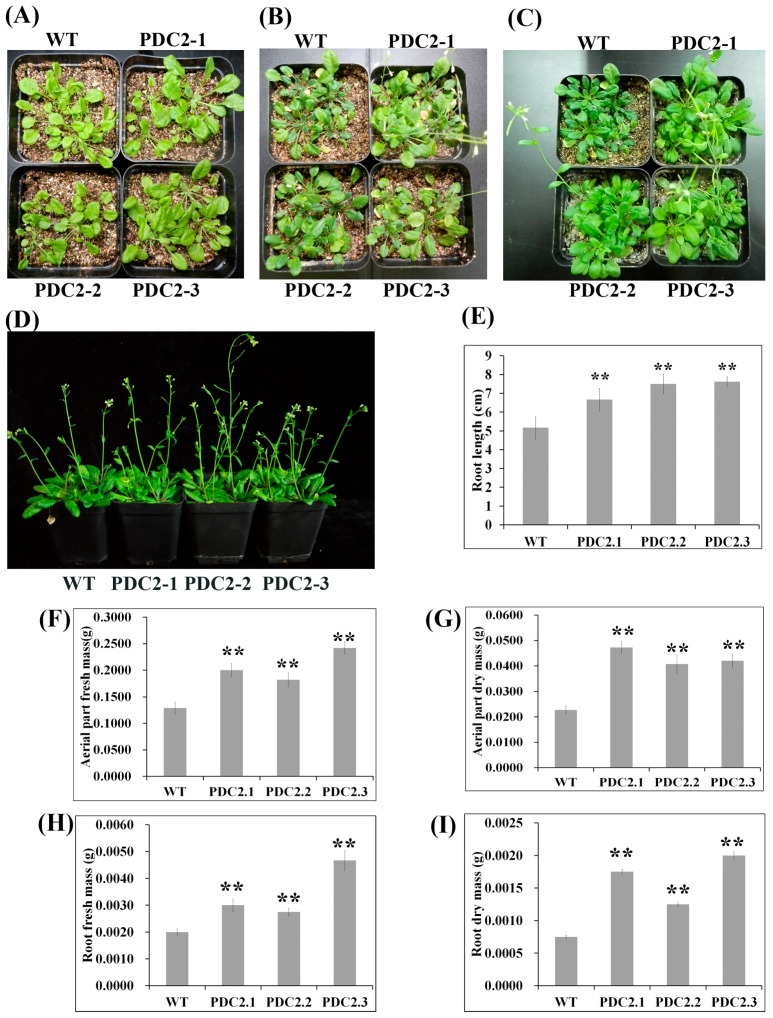
Five-week-old plants of transgenic *Arabidopsis* PDC2-1, PDC2-2, PDC2-3, and wild-type (WT) were used to detect waterlogging tolerance. (**A**) Before waterlogging assay; (**B**) waterlogging for two weeks; (**C**) growth recovery for one week after waterlogging stress; (**D**) plant growth under normal condition for 56 d (CK); (**E**) root length of C; (**F**) aerial part fresh mass of C; (**G**) aerial part dry mass of C; (**H**) root fresh mass of C; and (**I**) root dry mass of C. For E, F, G, H, and I, each column is the average of three replicates and each replicate has 36 plants. Bars indicate SD. Double asterisks indicate significant differences compared with WT line values (** *p* < 0.01).

Functional analysis of *AdPDC2* was investigated by its ectopic expression in *Arabidopsis*. The *35S: AdPDC2* transgenic *Arabidopsis* was obtained through hygromycin screening and the ectopic expression of *AdPDC2* was confirmed by qRT-PCR analysis from three independent transformation events (Figure S3). T_3_ seedlings of *A. thaliana* transgenic lines and wild type (WT) plants aged five weeks were used for waterlogging stress experiments. The transgenic *AdPDC2* lines had a higher waterlogging stress tolerance than WT plants after two weeks of waterlogging stress (Figure 3B). After recovery for one week, the growth of the transgenic *AdPDC2* lines was much better than that of the WT plants (Figure 3C). The root length (Figure 3E), aerial part fresh mass (Figure 3F), and dry mass (Figure 3G), root fresh mass (Figure 3H), and dry mass (Figure 3I) of the transgenic lines overexpressing the kiwifruit *AdPDC2* gene were significantly higher than those of WT plants after one week of growth recovery. Under normal conditions (Control check, CK), the WT and transgenic plants grew well and showed no significant difference in phenotypes (Figure 3D). These results showed that transgenic *Arabidopsis* overexpression of kiwifruit *AdPDC2* could enhance resistance to waterlogging stress.

### 2.3. Overexpression of the AdPDC2 Gene Enhances Resistance to Heat Stress

**Figure 4:**
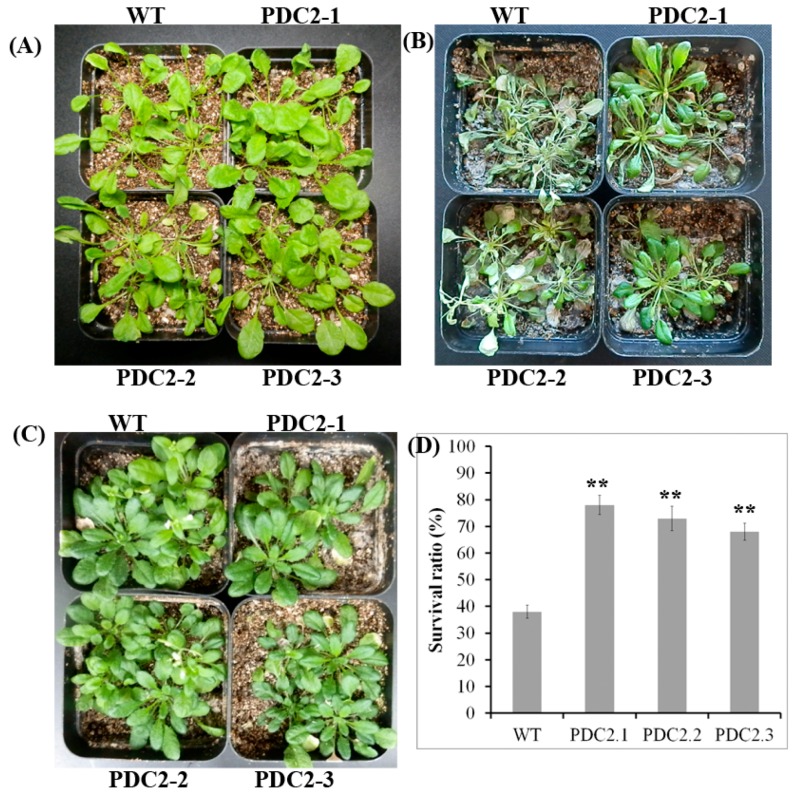
Phenotype of *AdPDC2* transgenic *Arabidopsis* and WT lines under heat stress. (**A**) The five-week-old transgenic and WT plants were grown in nutrient soil before heat stress; (**B**) The seedlings were kept at 37 °C for three days, and then recovery for one week; (**C**) The seedlings were planted in normal growth conditions for 45 d (CK); (**D**) Survival rate of plants in B after heat stress. For D, each column is the average of three replicates and each replicate has 36 plants. Bars indicate SD. The data presented are mean ±SD; Double asterisks indicate significant differences compared with control line values (** *p* < 0.01).

Five-week-old transgenic *Arabidopsis* plants were examined under heat stress to determine whether the overexpression of *AdPDC2* in *Arabidopsis* enhanced heat resistance (Figure 4). The results showed that the phenotype of overexpression lines displayed an outstanding tolerance after treatment at 37 °C for three days, followed by recovery for one week. The survival rate of the *AdPDC2* lines was significantly higher than that of the control lines. The WT and the transgenic plants grew well and showed no significant difference in phenotypes under normal conditions (CK). These results showed that overexpression of *AdPDC2* enhanced resistance to heat stress at the seedling stage.

### 2.4. Overexpression of the AdPDC2 Gene Could not Enhance Resistance to Salinity and Osmotic Stresses

Regarding the salt stress assays, there was no difference in the germination rates between the WT and transgenic *Arabidopsis* lines grown on the Murashige and Skoog (MS) medium and MS medium supplemented with NaCl (100 mM and 200 mM) (Figure S4). All transgenic and WT seedlings had a similar appearance and root lengths were not significantly different after treatment with the MS medium containing 100 mM NaCl. These results suggested that overexpression of the *AdPDC2* gene did not regulate seed germination and plant tolerance to salt stress at the seed germination and seedling stages.

Seeds of WT and transgenic *Arabidopsis* lines were germinated in MS supplemented with 100 mM or 300 mM mannitol, and the result showed that there were no differences in seed germination (Figure S5A,B). After the transgenic and WT *Arabidopsis* seedlings were treated with 300 mM mannitol, all of the transgenic and WT seedlings had a similar appearance (Figure S5C), and the root lengths were not significantly different (Figure S5D). These results suggested that overexpression of the *AdPDC2* gene did not enhance the resistance to mannitol stress at the seed germination and seedling stages.

## 3. Discussion

Many studies have been performed across a wide range of species in response to waterlogging, submergence, and hypoxia, with results showing that low O_2_ induced anaerobic metabolism module gene expression including amylases, sucrose synthase, phosphofructokinase, PDC, ADH, lactate dehydrogenase, and so on [9]. Waterlogging or submergence caused O_2_ deprivation in the soil [10,11]. Several reviews have summarized that three of the five ethylene response factor (ERF) VIIs genes were constitutively expressed (RAP2.12, RAP2.2, and RAP2.3) and further upregulated by darkness or ethylene in *A. thaliana*, and the other two ERFVIIs genes (HYPOXIA RESPONSIVE ERF1/2) were greatly enhanced at the transcriptional and translational levels by O_2_ deprivation [9,12,13]. RAP2.12 was re-localized from the plasma membrane to the nucleus as O_2_ concentrations declined, with the increased accumulation of hypoxia-responsive mRNAs, including *PDC1* and *hypoxia-responsive ERF1*/*2* (*HRE1*/*2*) [9].

Exposure of the roots to hypoxic conditions substantially increased the activities of two ethanol fermentation enzymes—ADH and PDC—in wheat [14], barley, and rice [15]. Two *PDC* and two *ADH* genes were induced in kiwifruit after waterlogging treatment using Illumina sequencing technology [4]. These results showed that ethanolic fermentation was classically associated with flooding tolerance. *PDC* and *ADH* are the two key enzymes of ethanolic fermentation. Waterlogging induced cotton *PDC* gene expression in both roots and shoots [16]. Transgenic *Arabidopsis* overexpressing *ADH* or *PDC* enhanced resistance to low oxygen conditions in roots [17,18]. In *Arabidopsis*, *AtPDC1* was strongly induced by anoxia. The *pdc1* mutant was more susceptible to anoxia, indicating that the *AtPDC1* gene of *Arabidopsis* was required during anoxia [19]. Furthermore, both *PDC1* and *PDC2* among the *PDC* gene family of *Arabidopsis* were significantly induced for expression during hypoxia and anoxia, and have been demonstrated to play an important role in submergence tolerance through mutant and transgenic experiments [2,20]. The expression of *PDC1*, *PDC2,* and *PDC4* was also strongly upregulated during flooding, which improved tolerance under long-term anoxia in rice [21,22]. We have previously reported that waterlogging significantly induced the expression of the *AdPDC1* gene in kiwifruit and the overexpression of the *AdPDC1* gene in *Arabidopsis* enhanced resistance to waterlogging [5]. In this study, another *PDC* gene named *AdPDC2* was investigated and the results were the same as for the *AdPDC1* gene, where the expression of the *AdPDC2* gene was significantly induced by waterlogging in kiwifruit (Figure 2) and the transgenic *Arabidopsis* overexpressing *AdPDC2* gene enhanced resistance to waterlogging (Figure 3). Thus, assembling *Arabidopsis AtPDC1* and *AtPDC2* [2,20], both kiwifruit *PDC* genes play key roles in waterlogging resistance. The expression of the kiwifruit *AdPDC1* gene was downregulated by ABA, and transgenic *Arabidopsis* overexpressing the kiwifruit *AdPDC1* gene inhibited seed germination and root length under ABA treatment, suggesting that ABA might negatively regulate the *AdPDC1* gene under waterlogging stress [5]. However, ABA could not induce the expression of *AdPDC2* in *A. deliciosa* (Figure 2), indicating that the *AdPDC2* gene could be regulated by other signal transduction pathways under waterlogging stress.

Heat, drought, and low temperatures are also major abiotic stresses with adverse effects on plant growth and productivity [23,24]. The expression of *HbPDC1* was increased in the bark and leaves of the rubber tree after treatment with low temperatures [25]. *AtPDC1* was induced by ABA, cold, salinity, mannitol, wounding, and paraquat in *Arabidopsis* [19]. However, the increased expression of *AtPDC1* was lower than during waterlogging stress. These results suggested the involvement of ethanolic fermentation in response to abiotic stress in plants, as indicated both at the transcriptional level and by the accumulation of ethanolic fermentation products. Overexpression of *PDC1* in *Arabidopsis* enhanced the low-temperature sweetening tolerance in transgenic potatoes [26]. A previous report showed that low temperatures and drought could not induce expression of the *AdPDC1* gene in kiwifruit, but salt and heat stress could induce the expression of *AdPDC1* [5]. However, overexpression of the *AdPDC1* gene in transgenic *Arabidopsis* did not enhance resistance to mannitol, cold, and salt stress [5]. In this study, the expression patterns of *AdPDC2* in kiwifruit after treatment with salt, heat, low temperature, and drought stress (Figure 2) were similar to those of *AdPDC1*. NaCl and heat stress could induce the expression of *AdPDC2*. However, the increased expression was lower than during waterlogging stress. Furthermore, transgenic *Arabidopsis* overexpressing the kiwifruit *AdPDC2* gene could enhance resistance to heat stress in seedlings (Figure 4), suggesting that the *AdPDC2* gene plays a key role in resistance to heat and waterlogging stress. Overexpression of kiwifruit *AdPDC2* in transgenic *Arabidopsis* could not enhance resistance to salt stress (Figure S4) at the seed germination and early seedling stages, suggesting the involvement of ethanolic fermentation in response to salt stress, as indicated by the transcriptional level. Low temperature and drought stress could not induce the expression of *AdPDC2* in kiwifruit. Overexpression of kiwifruit *AdPDC2* in transgenic *Arabidopsis* could not enhance resistance to mannitol stress (Figure S4) at the seed germination and early seedling stages, indicating that low temperature and drought stress did not involve an ethanolic fermentation response. Low temperature and drought stress did not involve ethanolic fermentation in kiwifruit, with the exception of waterlogging, salinity, and heat stress. These results showed that different *PDC* genes have various function characterizations in plants due to nucleotide and amino acid sequence differences. Functional analysis of more *PDC* genes is indispensable in the same and different species.

*Arabidopsis AtPDC1* and *AtPDC2* genes were expressed in all organs. The highest expression of *AtPDC1* was observed in the imbibed seeds of *Arabidopsis*, and then in silique [19]. The expression of *AtPDC2* was higher in the roots, shoots, flowers, siliques, and seeds than in the seedlings [19]. The tissue expression patterns suggested that different *HbPDC* isoforms perform major functions in different tissues: *HbPDC1* in leaves and shoots, *HbPDC2* and *HbPDC3* in bark, and *HbPDC4* in the latex and female flowers [25]. In petunias, *PDC2* is highly and exclusively expressed in the anthers and pollen, and an analysis of the *pdc2* mutant phenotype indicated the participation of *PDC2* in pollen tube elongation [27]. Additionally, the rice *PDC3* gene is specifically expressed in pollen and plays a role in aerobic alcoholic fermentation in mature pollen [28,29]. In this study, the expression levels of *AdPDC2* in the reproductive organs were much higher than those in the nutritive organs, suggesting that the kiwifruit *AdPDC2* gene plays a role in aerobic alcoholic fermentation in the reproductive organs.

## 4. Materials and Methods

### 4.1. Plant Materials and Growth Conditions

To allow the analysis of tissue-specific gene expression, different kiwifruit (*A. deliciosa* var deliciosa “Jinkui”) plant tissues, including the root, stem, leaf, anthocaulus, petal, pistil, calyx, ovary, stamen, and fruitlet (20 days after full blossom, DAFB) were collected from the Institute of Botany, Jiangsu Province and Chinese Academy of Sciences (32°18′ N118°52′ E). Different kiwifruit tissues were snap frozen in liquid nitrogen and stored −80 °C for later experiments. For treatments with ABA, heat, and low temperature (4 °C), plants were sub-cultured in vitro in Murashige and Skoog (MS) media supplemented with 6-benzylaminopurine (6-BA, 3.0 mg·L^−1^) and naphthalene acetic acid (NAA, 0.2 mg·L^−1^) under conditions of a 16 h light (23 ± 1 °C)/8 h dark (23 ± 1 °C) cycle. For treatments with waterlogging, salt, and drought stress, seedlings from a two-year-old *A. deliciosa* (Jinkui) cutting were grown in nutrient soil in a greenhouse with an air temperature of 25–28 °C during the day and 20–25 °C during the night.

*A. thaliana* (ecotype Columbia, Col-0,) seedlings were surface sterilized and planted in half MS medium containing 3% sucrose and 0.8% (*w*/*v*) agar. Plates were incubated at 4 °C for 2 days and transferred to a chamber at 22/20 °C (day/night), with a photoperiod of 16/8 h (day/night) for 7 days, then transplanted into nutrient soil and planted in the same conditions with a photosynthetic photon flux density of 180 μmol/m^2^·s.

### 4.2. Treatment of A. deliciosa with Abiotic Stresses

The stress treatments were performed in kiwifruit as described previously in [5], and non-treated plants were used as the control (CK). For waterlogging stress, the pots were flooded for 0, 24, 48, and 96 h, respectively, and roots were harvested and stored at −80 °C. For salt and ABA treatment, seedlings were soaked in 200 mM NaCl or 0.01 mM ABA (Sigma) for 4, 12, and 48 h. For drought stress, plants were dried for 14 days. For freezing stress, seedlings were grown at 4 °C for 4, 12, and 48 h. For heat stress, seedlings were grown at 48 °C for 2 and 4 h, then at 23 ± 1 °C for another 6 h. The leaves from the salt, ABA, drought, freezing, heat stress, and control treatments were harvested and stored at −80 °C. Each experiment was performed three times with each replication containing 15 plants.

### 4.3. Total Ribonucleic Acid (RNA) Isolation and cDNA Synthesis

Total RNA was isolated as described in [30]. Reverse-transcription of mRNA was performed with 1.0 μg mRNA using a PrimeScript^TM^ RT Reagent Kit with a gDNA eraser (Perfect Real Time, TaKaRa, Cat. #RR047Q, Dalian, China).

### 4.4. Cloning and Sequence Analysis of AdPDC2

Complete CDS of *AdPDC2* was isolated by RT-PCR according to the sequence comp110797-co-seq1 obtained by Illumina sequencing [4]. Specific primers for the amplification of full-length genes are listed in the Supporting Information Table S1. The gene was cloned into pMD19-T vector (TaKaRa, Dalian, China) and sequenced. The nucleotide and deduced amino acid sequences of two kiwifruit *PDC* genes were compared using the BLAST program. Protein sequence alignment was performed using the BioEdit software (v 7. 0. 5, Ibis Therapeutics, Carlsbad, CA, USA). Molecular weight and isoelectric point (PI) were obtained using online analysis software (Swiss Institute of Bioinformatics, Geneva, Swiss) [31].

### 4.5. Real-Time Quantitative Reverse Transcription Polymerase Chain Reaction (qRT-PCR) Assay for AdPDC2 Gene Expression

QRT-PCR was carried out on an Applied Biosystems 7300 Real Time PCR System as per the method reported by Zhang et al. [5]. Each PCR reaction contained 10 μL SYBR^®^ Premix Ex Taq™ (Perfect Real Time, TaKaRa, code: DRR041A), 0.3 μL (10 pM) of each primer (Table S1), 8.4 μL sterile double-distilled water, and 1 μL of each reverser transcribed cDNA product. To normalize the relative gene expression levels in kiwifruit, actin was used as the internal control [32]. The *AdPDC2* and *AdActin* genes were amplified using normal PCR, and a single PCR fragment was obtained, cloned, and sequenced to confirm the accurate fragment, suggesting that the primers were suitable for qRT-PCR analysis. Technical triplicates were done for each biological replicate. The relative fold change of expression was calculated as per Zhang et al. [5]. The statistical significance was performed using SPSS version 17.0 statistical software (SPSS Corp., Chicago, IL, USA).

### 4.6. Generation of AdPDC2-Overexpressing Arabidopsis Plants

The complete CDS of the *AdPDC2* was cloned into pCAMBIA1301 and driven by a CaMV 35S promoter. The *AdPDC2* gene was transformed into *Arabidopsis* ecotype Columbia plants (Col-0) using the flower dip method via *Agrobacterium* mediated transformation [33]. The transgenic *Arabidopsis* lines were selected in the half-strength MS medium containing hygromycin (20 mg/mL). Survival transgenic plants were grown in the greenhouse at 22 °C under long-day (16-h photoperiod) exposure. Homozygous T_3_ seeds were used for stress tolerance assays. QRT-PCR was performed using the primers AF2 and AR2 (Table S1) to verify the transgenic lines. Control reactions to normalize qRT-PCR were completed using *AtUBQ10* as the house-keeping gene with sequences derived from *Arabidopsis* [34].

### 4.7. Analysis of Transgenic Lines for Tolerance to Waterlogging, NaCl, and Mannitol

For waterlogging, salinity, and mannitol stresses, assays were performed as described previously in [5]. Five-week-old *A. thaliana* homozygous T_3_ seedlings from transgenic lines and WT seedlings were waterlogged for two weeks, followed by a one-week recovery. The phenotypic changes of seedlings were observed. After a one-week recovery, the seedlings root length, aerial part fresh mass and dry mass, root fresh mass and dry mass were measured. The experiments were repeated three times. To analyze the salinity and mannitol stress tolerance, sterilized seeds were planted on MS agar medium supplemented with NaCl (100 mM and 200 mM) or mannitol (100 mM and 300 mM), respectively. The germination ratio was determined at 7 d, and the phenotypic traits of the seedlings were observed at 10 d. Each experiment was repeated three times with each replicate containing at least 50 seeds. Additionally, sterilized seeds were planted on MS agar medium for 4 d, and then transferred to MS medium containing NaCl (100 mM) or mannitol (300 mM), respectively. The root lengths of the seedlings were measured and phenotype traits were observed after 7 d treatment. Experiments were performed for three replicates of 30 seedlings. For the heat stress assay, five-week-old transgenic and WT plants were kept at 37 °C for 3 d, followed by recovery for one week. The phenotypic traits were observed one week after recovery. The survival rate of the plants was tested. The experiments were repeated three times. Statistically significant differences were calculated with SPSS version 17.0 statistical software (SPSS Corp., Chicago, IL, USA).

## 5. Conclusions

In summary, waterlogging and heat stresses could induce the expression of *AdPDC2* in kiwifruit, while transgenic *Arabidopsis* overexpressing the kiwifruit *AdPDC2* gene could enhance resistance to waterlogging and heat stress at the seedling stage, suggesting that the *AdPDC2* gene may be important in waterlogging and heat stress responses in kiwifruit. It is known that waterlogging and heat stress are accompanied in plants. Kiwifruit *AdPDC2* has the double function of resistance to waterlogging and heat stress, so the *AdPDC2* gene has great application potential in plant breeding in the future.

## Supplementary Materials

Supplementary materials can be found at www.mdpi.com/1422-0067/18/11/2377/s1.
