# Supplementary material for: Functional Characterization of Waterlogging and Heat Stresses Tolerance Gene Pyruvate decarboxylase 2 from Actinidia deliciosa
Source: Int J Mol Sci. 2017 Nov 9;18(11):2377. doi: 10.3390/ijms18112377 (PMC5713346; doi:10.3390/ijms18112377)
Supplement: Supplementary file 1 [file ijms-18-02377-s001.pdf]

Table S1 - Sequence of primers used in this paper

gene	Primer names	Sequence5'-3'	usage
AdPDC2	AF1	***GGATCC***ATGGCAACCGCCACCCA	cDNA cloning and construction of plant Binary expression vector for transformation
	AR1	***GGTACC***CTATTGAGGATTAGGAG	
	AF2	TCATAATGGTGAAGGCAAATGCTG	Expression of *AdPDC2* in kiwifruit
	AR2	GCTCGTGTCGTCCTTGTGAAC	
AdActin	Act-F	TGCATGAGCGATCAAGTTTCAAG	*Actinidia deliciosa* housekeeping gene
	Act-R	TGTCCCATGTCTGGTTGATGACT	
AtUBQ10	AtUBQ10-F	GGACCAGCAGCGTCTCATCTTCGCT	*Arabidopsis* housekeeping gene
	AtUBQ10-R	CTTATTCATCAGGGATTATACAAG	

Notes: Italics base pairs indicated the restriction point;

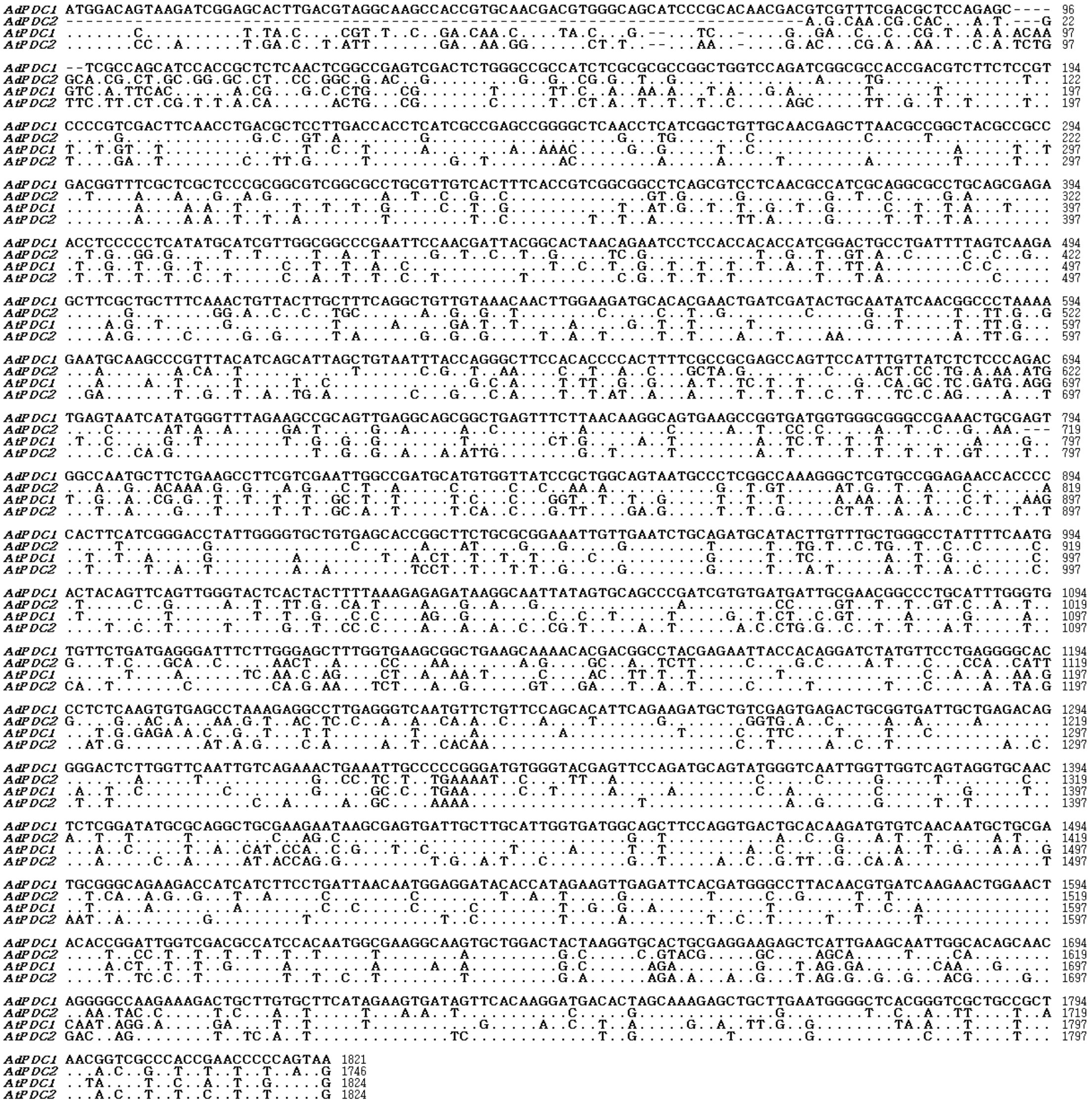

Figure S1 Nucleotide sequence alignment of *A. deliciosa AdPDC1* and *AdPDC2* with *A. thaliana AtPDC1* and *AtPDC2*. *AtPDC1*: *A. thaliana PDC1* (GenBank accession no. NM_124878); *AtPDC2*: *A. thaliana PDC2* (GenBank accession no. NM_119461). *AdPDC1*: *A. deliciosa AdPDC1* (GenBank accession no. KU095879). Dots indicate that the amino acid residues were conserved in each group, and transverse lines suggest gaps in nucleotide sequences.

```
AdPDC1   MDSKIGALDVGKPPCNDVGSIPHNDVVSTLQSSP-ASTALNSAESTLGRHLARRLVQIGA 59
AdPDC2   -------------------------MATAT.IGTASPAPFPGGTW.....V........V 35
AtPDC1   ..T...SI.DC..TNG..C.PTNGT.ATIHN.V.SSAITI.YCDA............A.V 60
AtPDC2   ..T...SI.ACN.TNH.I.GP.NGG.STVQNT..LH..TVSPCDA....Y......E..V 60

AdPDC1   TDVFSVPVDFNLTLLDHLIAEPGLNLIGCCNELNAGYAADGFARSRGVGACVVTFTVGGL 119
AdPDC2   .......G....A...........S.V..............Y..A............... 95
AtPDC1   .......G..........M...D..................Y.................. 120
AtPDC2   .......G..............N.K................Y.................. 120

AdPDC1   SVLNAIAGACSENLPLICIVGGPNSNDYGTNRILHHTIGLPDFSQELRCFQTVTCFQAVV 179
AdPDC2   .........Y.....V...................................A...A.... 155
AtPDC1   .........Y.............................................Y.... 180
AtPDC2   .........Y..................................T.......A.......I 180

AdPDC1   NNLEDAHELIDTAISTALKECKPVYISISCNLPGLPHPTFRREPVPFVISPRLSNHMGLE 239
AdPDC2   ...D................S..A..........I.....A.....YFLAQNV..QL... 215
AtPDC1   ...D....Q..K........S......V....AAI..H..S.D....SLA.....K.... 240
AtPDC2   ....E...............S............AI.L...S.H....MLPMKV..QI..D 240

AdPDC1   AAVEAAAEFLNKAVKPVMVGGPKLRVANASEAFVELADACGYPLAVMPSAKGLVPENHPH 299
AdPDC2   T................L.......-.K.QK..M.........I.....G......H... 274
AtPDC1   .....TL....................K.CD........S..A..M......F...H... 300
AtPDC2   .................L.....M...K.AD........S..G.........Q...H.K. 300

AdPDC1   FIGTYWGAVSTGFCAEIVESADAYLFAGPIFNDYSSVGYSLLLKRDKAIIVQPDRVMIAN 359
AdPDC2   ...........SY.G.........V.V...............I.KE..V....N..T.G. 334
AtPDC1   ...........P..S.........I...................KE...V.....ITV.. 360
AtPDC2   ...........A................................KE..........T.G. 360

AdPDC1   GPAFGCVLMRDFLGALVKRLKQNTTAYENYHRIYVPEGHPLKCEPKEALRVNVLFQHIQK 419
AdPDC2   ..SL.W.F.A...T..A.K..K.S..L...R..F..P.IA..R.ND.P....I..K...E 394
AtPDC1   ..T...I..S..FRE.S..V.R.E.........F....K.....SR.P....TM...... 420
AtPDC2   .........K...SE.A..I.H.N.S............K..RDN.N.S...........N 420

AdPDC1   MLSSETAVIAETGDSWFNCQKLKLPPGCGYEFQMQYGSIGWSVGATLGYAQAAKNKRVIA 479
AdPDC2   ..GGD.................H..EN..F........................D..... 454
AtPDC1   .........................K..........................SPE...L. 480
AtPDC2   .....S..L................E..........................MP.R.... 480

AdPDC1   CIGDGSFQVTAQDVSTMLRCGQKTIIFLINNGGYTIEVEIHDGPYNVIKNWNYTGLVDAI 539
AdPDC2   .............I...I..A.RS.................................... 514
AtPDC1   F.........V..I.....N........................................ 540
AtPDC2   .................I....................................AF.E.. 540

AdPDC1   HNGEGKCWTTKVHCEEELIEAIGTATGAKKDCLCFIEVIVHKDDTSKELLEWGSRVAAAN 599
AdPDC2   .........A..RT..Q.A...A...EVH..S.....I..................S... 574
AtPDC1   .....N...A..RY....V...T...TE...........L................S... 600
AtPDC2   .........A..R.....VK..N...NEE.ESF.......................S... 600

AdPDC1   GRPPNPQ 606
AdPDC2   S...... 581
AtPDC1   S...... 607
AtPDC2   S...... 607
```

Figure S2. Amino acid sequence identity of *A. deliciosa* AdPDC1 and AdPDC2 with A. thaliana *AtPDC1* and AtPDC2. AtPDC1: *A. thaliana* PDC1 (GenBank accession no. NP_200307); AtPDC2: *A. thaliana* PDC2 (GenBank accession no. NP_195033). AdPDC1: *A. deliciosa* AdPDC1 (GenBank accession no. ALX37952). Dots indicate that the amino acid residues were conserved in each group, and transverse lines suggest gaps in amino acid sequences.

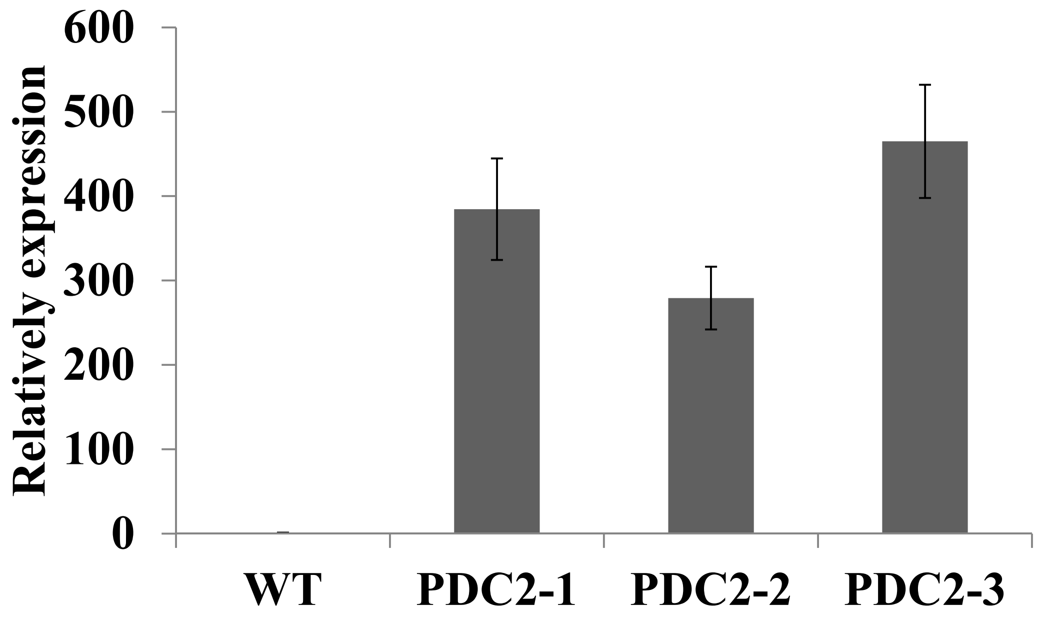

Fig. S3 Molecular confirmation of MdPDC2 transgenic Arabidopsis plants using qRT-PCR WT: Wild type; PDC2-1, PDC2-2 PDC2-3: transgenic lines. The data presented are the mean ± SD.

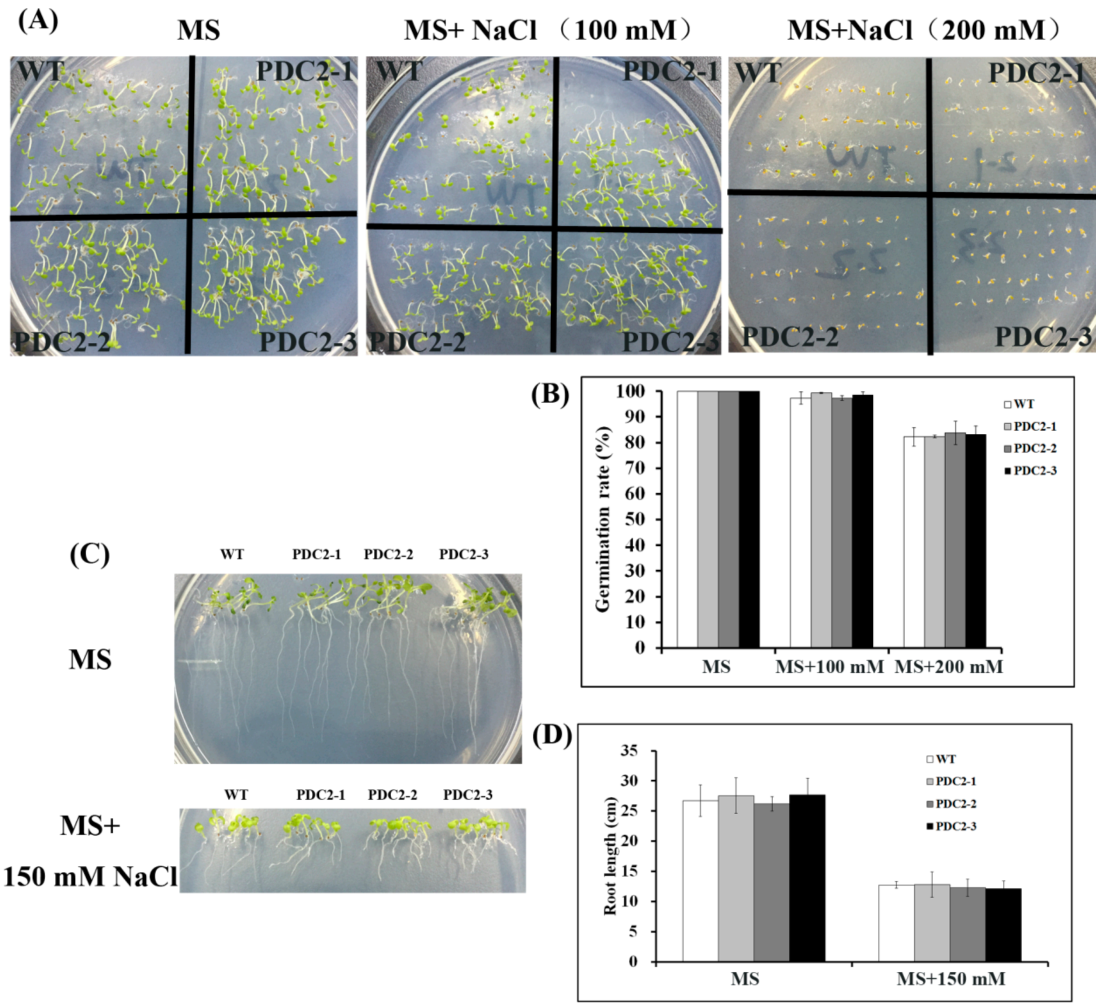

Figure S4 Transgenic *Arabidopsis* overexpression of *AdPDC2* (PDC2-1, PDC2-2 and PDC2-3) and wild-type (WT) were used to detect the salinity tolerance. A: Photographs were taken 10 days after seed germination of WT and transgenic *Arabidopsis* plants in MS medium containing various concentrations of NaCl (0, 100 mM and 200 mM). B: Germination rates in WT and transgenic plants grown in MS (control) or MS supplemented with various concentrations of NaCl (100 mM and 200 mM) for 7 days. 50 seeds of each line were grown for each experiment. C: Comparison of root length between transgenic and WT lines under NaCl stress. Four day-old seedlings of WT and transgenic seedlings which were germinated just from MS agar medium were transferred to MS medium containing NaCl (150 mM). The phenotype (C) and root length (D) were measured after 7 days. The experiments were carried out on three replicates of 30 seedlings. The results from one set of experiments are shown. Results are presented as mean ± SD from three independent experiments.

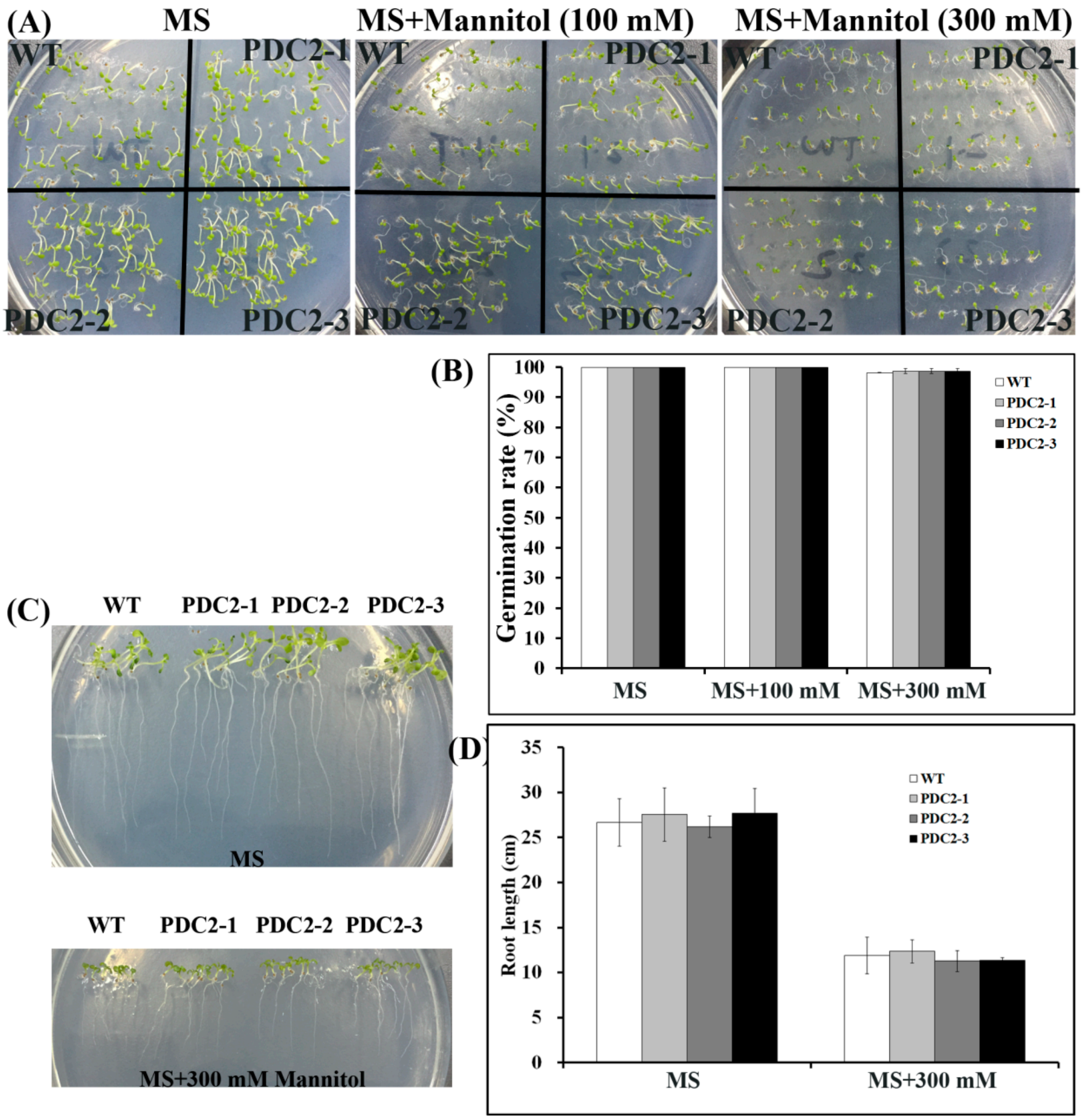

Figure S5 Transgenic *Arabidopsis* overexpression of *AdPDC2* (PDC2-1, PDC2-2 and PDC2-3) and wild-type (WT) were used to detect the mannitol tolerance. Photographs were taken 10 days after seed germination of WT and transgenic *Arabidopsis* plants in MS medium, MS medium containing 100 mM mannitol and 300 mM mannitol (A). B: Germination rates in WT and transgenic plants grown in MS (control) or MS supplemented with various concentrations of mannitol (100 mM and 300 mM) for 7 days. 50 seeds of each line were grown for each experiment.C: Comparison of root length between transgenic and WT lines under mannitol stress. Four day-old seedlings of WT and transgenic seedlings which were germinated just from MS agar medium were transferred to MS medium containing mannitol (300 mM). The phenotype (C) and root length (D) were measured after 7 days. The experiments were carried out on three replicates of 30 seedlings. The results from one set of experiments are shown. Results are presented as mean ± SD from three independent experiments
